# Exosomes Derived from Dental Pulp Stem Cells Show Different Angiogenic and Osteogenic Properties in Relation to the Age of the Donor

**DOI:** 10.3390/pharmaceutics14050908

**Published:** 2022-04-21

**Authors:** Giulia Brunello, Federica Zanotti, Martina Trentini, Ilaria Zanolla, Elham Pishavar, Vittorio Favero, Riccardo Favero, Lorenzo Favero, Eriberto Bressan, Massimo Bonora, Stefano Sivolella, Barbara Zavan

**Affiliations:** 1 Department of Neurosciences, School of Dentistry, University of Padua, 35128 Padua, Italy; giulia.brunello@unipd.it (G.B.); rickyfavero@mns.com (R.F.); lorenzo.favero@unipd.it (L.F.); eriberto.bressan@unipd.it (E.B.); stefano.sivolella@unipd.it (S.S.); 2 Department of Oral Surgery, University Hospital of Düsseldorf, 40225 Dusseldorf, Germany; 3 Department of Translational Medicine, University of Ferrara, 44121 Ferrara, Italy; federica.zanotti@unife.it (F.Z.); trentini.martina.94@gmail.com (M.T.); znllri@unife.it (I.Z.); elham.pishavar@unife.it (E.P.); 4 Unit of Maxillofacial Surgery and Dentistry, University of Verona, 37129 Verona, Italy; vittorio_favero@yahoo.it; 5 Department of Medical Sciences, University of Ferrara, 44121 Ferrara, Italy; massimo.bonora1@unife.it

**Keywords:** exosomes, miRNA, bone regeneration, dental pulp, mesenchymal stem cells, regenerative medicine

## Abstract

Craniofacial tissue reconstruction still represents a challenge in regenerative medicine. Mesenchymal stem cell (MSC)-based tissue engineering strategies have been introduced to enhance bone tissue repair. However, the risk of related complications is limiting their usage. To overcome these drawbacks, exosomes (EXOs) derived from MSCs have been recently proposed as a cell-free alternative to MSCs to direct tissue regeneration. It was hypothesized that there is a correlation between the biological properties of exosomes derived from the dental pulp and the age of the donor. The aim of the study was to investigate the effect of EXOs derived from dental pulp stem cells of permanent teeth (old donor group) or exfoliated deciduous teeth (young donor group) on MSCs cultured in vitro. Proliferation potential was evaluated by doubling time, and commitment ability by gene expression and biochemical quantification for tissue-specific factors. Results showed a well-defined proliferative influence for the younger donor aged group. Similarly, a higher commitment ability was detected in the young group. In conclusion, EXOs could be employed to promote bone regeneration, likely playing an important role in neo-angiogenesis in early healing phases.

## 1. Introduction

In maxillofacial surgery repair and functional regeneration of large bony defects secondary to tumor resections, congenital deformities, or trauma represent a challenge. Several scaffolds of different origin are widely used to treat these bone defects, but several problems often occur due to the lack of vascularization inside the scaffolds. In this contex in the last 20 years of research great attention has been devoted to the development tissue engineering strategies [1,2,3,4]. These are based on the use of scaffolds, that act as a guide for tissue regeneration, of cells and of growth factors, that are supposed to foster the healing process [5,6]. In this view several stem cell types have been proposed for tissue regeneration. The most studied are mesenchymal stem cells (MSCs), including dental pulp stem cells (DPSCs), that have been demonstrated to promote bone tissue regeneration [7,8,9,10,11]. Their ability to start an unpredictable commitment in vivo is a risk that contributes to their limited clinical applications. Recently the researchers have focused their attention on alternative treatment options in tissue engineering, such as cell-free-based therapy. The secretome of individual cells is definitely a hot topic in this context. In particular, exosomes (EXOs) are the principal bioactive components owing to their high informative content formed by protein, mRNA and microRNA (miRNA). Compared with cell-based applications, exosomes show key advantages over cell-based therapy, i.e., resolving the problems associated with the commitment risk, the immune incompatibility, emboli formation, tumorigenicity, and the transmission of infections that can be prevented.

In bone regeneration several articles confirm the potential role of miRNAs present inside the exosomes not only in the regeneration of new tissues but also in the significant decline in musculoskeletal function observed in older individuals [12,13,14,15]. Moreover, their particular miRNA content (e.g., miR-206, miR-27a and miR-196a), when transferred into osteoblasts, activates the gene expression of osteogenic-related genes, such as ALP and RUNX2, that induce the osteogenic commitment. Takeuchi et al. indicated that MSC-derived EXOs enhance osteogenesis and angiogenesis, when implanted in calvaria bone defects in rats [16]. In addition to miRNAs, it has been shown that tissue-specific transcription factors control cell lineage commitment trough the activation of downstream targets related to a cell’s type [17,18]. Indeed, it has been demonstrated that the combination of exosomes derived from osteoblasts and adipocyte exosomes may influence the differentiation of hMSCs into the respective lineage [17]. Furthermore, Yang et al. revealed the beneficial effect of non-invasive, light-driven approaches to improve the efficacy of exosome-based therapies [19]. Their results showed that blue light with a wavelength of 455 nm could stimulate angiogenesis capability of EXOs derived from human umbilical cord MSCs (UC-MSCs), both in vitro and in vivo, by increasing the levels of miR-135b-5p and miR-499a-3p [19]. Cellular communication within the bone marrow microenvironment is a dynamic process which requires to be further investigated [20,21]. Only recently it has been discovered that EXOs play a crucial role in this process [17]. Under a therapeutic point of view, we can conclude that exosomes show a great potential as a cell-free based tissue regeneration strategy, since these nano subcellular structures can overcome the problems associated to the use of living cells. Cheaper, more effective, and with no associated side effects, they represent the new frontiers for tissue regeneration. In this context the research is now focusing on discovering the best source of these natural nano tools, taking in mind the age of the donors; indeed, also in our previous study, cells derived from older donors were found to possess a lower proliferative ability in vitro as compared to DPSCs obtained from younger individuals [12,22,23]. Hence, our question is whether the properties of the exosomes are also related to the age of the cell donors, starting from the data currently available in the literature, showing that these cells produce exosomes with miRNA content promoting bone regeneration [24,25,26,27,28].

Therefore, the aim of the present study is to evaluate if there is any difference in the effect of EXOs derived from mesenchymal stem cells derived from old vs young donors. In this case we used dental pulp stem cells from permanent teeth as old donor group, and stem cells from exfoliated deciduous teeth (SHEDs) as younger donor group. Their biological activity was analyzed on bone marrow mesenchymal stem cells, which represent the main source of cells involved in bone regeneration.

## 2. Materials and Methods

### 2.1. Cell Culture

The hMSCs derived from bone marrow were purchased from ATCC (Manassas, VA, USA), Human dental pulp stem cells (namely: “old donor stem cells” from 15 to 30 years old) were purchased from Lonza, for the young donor group, stem cells derived from human exfoliated deciduous teeth stem cells (Creative bioarray) were utilized. Cells were cultured in Dulbecco’s Modified Eagle Medium (DMEM) (Lonza S.r.l., Milano, Italy) supplemented with 10% fetal bovine serum (FBS) (Bidachem S.p.A., Milano, Italy) and 1% penicillin/streptomycin (P/S) (EuroClone, Milan, Italy) to form complete DMEM (cDMEM) in T25 flasks. 

### 2.2. Exosomes Isolation from Stem Cells

Exosome isolation was performed from the growth medium of Dental Pulp Stem Cells (DPSCs) and exfoliated deciduous teeth cells (SHEDs), representing the old and young donor group. Therefore, the two exosome fractions will be hereafter called exoY (Young) and exoO (Old), respectively. About 1 × 10^7^ cells (passage 4) were cultured in complete DMEM (cDMEM) medium. For both extractions, cell medium supernatant was collected and centrifugated at increasing speed: 200 rpm for 10 min, 500 rpm for 10 min, and 2000 rpm for 20 min. After each centrifugation step, the supernatant was collected, and pellet was discharged. Finally, the supernatant was centrifugated at 100,000 rpm for 75 min and the pellet, which represents the exosome fraction, was resuspended in 1 mL of filtered PBS [29]. Exosomes from an entire T-175 flask (~50 μg) were dissolved in 500 μL of PBS (~100 ng/μL). For each test cells were treated with exosomes at a ratio of 1 μL: 50,000 cells. 

### 2.3. Characterization of Exosomes Citofluorimetro

The exosome size and concentration were determined using NanoSight (NS300, Malvern Instruments, Malvern, UK) [29]. Exosomes resuspended in phosphate buffered saline (PBS; EuroClone, Milano, Italy) were observed with blue laser (405 nm) and their movement under Brownian motion was captured for 60 s. NanoSight was used with a standard detection threshold of 3 and camera level set at 14 for all the experiments. Exosome concentration and size distribution profiles were determined by analyzing the captured video using NanoSight particle tracking software. All measurements were repeated 3 times. Superficial Markers has been evaluated by means of flow cytometry after incubation for 30 min with mouse anti-human CD63 APC, CD81 R-PE, in 500 µL of flow cytometry staining buffer. Attune NxT flow cytometer (Thermo Fisher Scientific, Waltham, MA, USA) has been used for the analysis. The presence of the same markers has been moreover detected with real time PCR. Total RNA was isolated from cells, 500 ng of total RNA for each sample was reverse-transcribed using an RT2 First Strand kit (Qiagen, Hilden, Germany). Real-time PCR was performed with a StepOnePlus™ Real-Time PCR System (Applied Biosystems™, Foster City, CA, USA), RT2 SYBR Green ROX FAST Master Mix (Qiagen, Hilden, Germany). Thermal cycling and fluorescence detection were as follows: 95 °C for 10 min, followed by 40 cycles of 95 °C for 15 s, and 60 °C for 1 min. At the end of each run, a melting curve analysis was performed using the following program: 95 °C for 1 min, 65 °C for 2 min with optics off, 65 °C to 95 °C at 2 °C/min with optics on.

### 2.4. Transmission Electron Microscopy (TEM)

For the visualization of exosomes, it was resorted to electronic microscopy as previously described [29]. Briefly, the exosome fraction, isolated as mentioned in Section 2.2, was fixed in a 2.5% glutaraldehyde/0.1 M sodium cacodylate buffer for 12 h at 4 °C. A solution of 1% OsO_4_/0.1 M sodium cacodylate buffer was used to maintain pH during fixation. The exosome fraction was then dehydrated by exposure to increasing concentrations of EtOH. Condensation of the sample was achieved with the use of epoxy resin (EPON™, Hexion, Houston, TX, USA). For the preparation of slides, ultrathin sections were prepared by cutting the fixed sample with ultramicrotome (LKB, Stockholm, Sweden), which were then stained with 1% uranyl acetate and 1% citrate solution. TEM microscope (Tecnai G12, FEI Company, Hillsboro, OR, USA) was used for sample visualization with acceleration voltage of 100 kV. Images were acquired by video camera (Tietz, Tietz Video and Image Processing Systems GmbH, Gauting, Germany) mounted on the microscope, and the imaging software TIA (FEI Company, Hillsboro, OR, USA).

### 2.5. Real-Time Polymerase Chain Reaction (qPCR)

In this case, qPCR was performed to analyze the commitment ability of the cells, the miRNA content inside the cells and the miRNA content of the exosomes (extracellular miRNA). Exosomal miRNA expression profiles were analyzed using miScript miRNA PCR Arrays (MIHS-001Z, Qiagen, Hilden, Germany) that investigated the expression of the 84 most abundantly expressed human miRNAs. The cDNA was synthesized from the total RNA using the miScript II Reverse Transcription Kit (Qiagen, Hilden, Germany), according to the user manual. The cDNA was pre-amplified with the miScript PreAMP PCR Kit, then mixed with QuantiTect SYBR Green PCR Master Mix, miScript Universal Primer, and RNase-free water. Real-time PCR was performed according to the user’s manual on inflammatory response and Autoimmunity RT2 profiler PCR Array (Qiagen, Hilden, Germany) or the primer reported on Table 1 with a StepOnePlus™ Real-Time PCR System (Applied Biosystems™, Foster City, CA, USA) using RT2 SYBR Green ROX FAST Master Mix (Qiagen, Hilden, Germany).

### 2.6. MTT Assay

To evaluate the possible cytotoxicity of the exosome fraction, we performed the MTT assay and observed the cell proliferation rate of MSCs at different time points [30,31]. For this purpose, MSCs were cultured both in exosome enriched medium and in normal conditions for 3, 7 and 10 days. At these time points, the culture medium was removed and cells were incubated at 37 °C for 3 h in 1 mL of MTT solution (0.5 mg/mL MTT in PBS). Intracellular MTT was then extracted with 0.5 mL of 10% dimethyl sulfoxide in isopropanol for 30 min at 37 °C and its optical density (O.D.) values were recorded in duplicate at 570 nm, in 200-μL aliquots using a multilabel plate reader (Victor 3, Perkin Elmer, Milan, Italy).

### 2.7. Population Time Doupling (PDT) Assay

To determine the different effect of exoO and exoY on cell proliferation, the growth of MSCs exposed to exosomes (exoO and exoY separately) was investigated by the population doubling time (PDT) assay. Briefly, 1.2 × 10^5^ MSCs were seeded into 6-well plates and exposed to whole fractions of exoO and exoY. After 3, 7 and 10 days from the beginning of the exposure, cells were detached, counted, and seeded again at the same density in a new 6-well plate. This was repeated until the cells reached p6.

### 2.8. ALP Activity Assay

The alkaline phosphatase (ALP) activity was measured to evaluate the initial differentiation of MSCs into preosteoblasts. MSCs were cultured in three different conditions: in presence of exoO, in presence of exoY and in standard medium. Intracellular and extracellular ALP activity was measured after 3, 6 and 9 days, with abcam’s Alkaline phosphates kit (colorimetric assay). The kit uses p-nitrophenyl phosphate (pNPP) as a phosphatase substrate which adsorbed at 405 nm when dephosphorylated by ALP. According to the manufacturer protocol, the culture medium from each sample group was collected and pooled together. At the same time, cells were washed with PBS and then homogenized with ALP Assay Buffer (300 μL in total for each group) and centrifuged at 13,000 rpm for 3 min to remove insoluble material. Different volumes of samples (medium and cells) were then added into 96-well plate, bringing the total volume in each well up to 80 μL with Assay Buffer. 80 μL of fresh medium was also utilized as sample background control. Thereafter, 50 μL of 5 mM pNPP solution was added to each well containing test samples and background control and incubated for 60 min at 25 °C, protecting the plate from the light. A standard curve of 0, 4, 6, 12, 16, and 20 nmol/well was generated from 1 mM pNPP standard solution bringing the final volume to 120 μL with Assay Buffer. All reactions were then stopped by adding 20 μL of Stop solution into each standard and sample reaction except the sample background control reaction. Optical density was read at 405 nm in a microplate reader (Victor, Perkin Elmer, Milan, Italy).

### 2.9. Quantification of Secreted Factors

Bio-Plex protein assay (Bio-Rad, Hercules, CA, USA) was used for the quantification of factors secreted by MSCs cells in different conditions. MSCs were grown in medium spiked with exoO, exoY and in standard medium as a control sample. The quantification was performed for (i) hepatocyte growth factor (HGF), (ii) basic fibroblast growth factor (b-FGF), (iii) vascular endothelial growth factor (VEGF), (iv) monocyte chemotactic protein-1 (MCP-1), (v) stromal cell-derived factor 1-alpha (SDF-1a), (vi) interleukin 1 receptor antagonist (IL-1ra), and (vii) macrophage colony-stimulating factor (M-CSF). After 3 days of culture, cells were washed twice with standard culture medium w/o FBS and the quantification was carried on following the manufacturer’s instructions [32].

### 2.10. Statistical Analysis

Each experiment was performed independently in triplicate. Statistical calculations were performed using GraphPad software (GaphPad, La Jolla, CA, USA). The mean values for quantitative data were compared applying non-parametric Kruskal-Wallis test for MTT assay.

Real-time PCR data were analyzed using Student’s unpaired *t*-test based on 2^−ΔΔCt^ values for each gene of the test group compared with that of the control group. Significance levels were calculated in comparison to the control condition and indicated as follows: *p* < 0.05, *p* < 0.01, *p* < 0.001.

Moreover, to calculate the population doubling (PD) of the cells from the PDT assay, we used the formula:

PDT = (T − T0)log2/logN_t_ − log_0_ (1)

where PDT represents the number of cell divisions that occur in each passage; N_t_ corresponds to cell number on the second day, and N_0_ is the initial seeding number of cells. To determine the Cumulative population doubling (CPD), the PDT level for each passage was calculated and added to the levels of the previous passages. The experiment was performed independently three times.

Lastly, data analysis for ALP assay was carried on as follows: O.D. values were normalized subtracting the value derived from the zero standards from all standards, samples and sample background control. The pNP standard curve was plotted to identify the pNP concentration in each sample. ALP activity of the test samples was calculated with the formula:

ALP activity (U/mL) = A/V/T(2)

where: A is the amount of pNP generated by samples (in μmol), V is the amount of sample added in the assay well (in mL), and T is the reaction times (in minutes).

## 3. Results

### 3.1. Morphological Characterization of Exososomes: Size, Distribution, Superficial Markers

**Figure 1:**
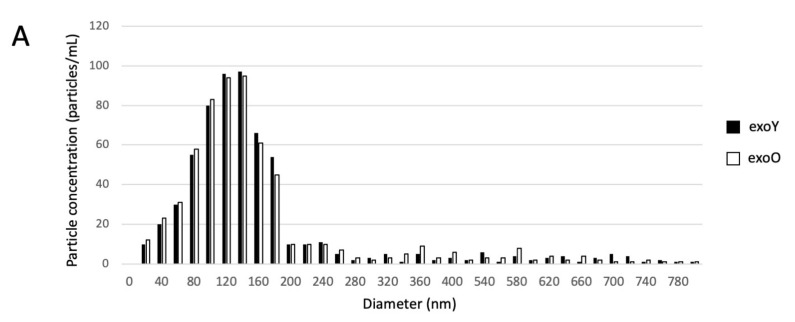

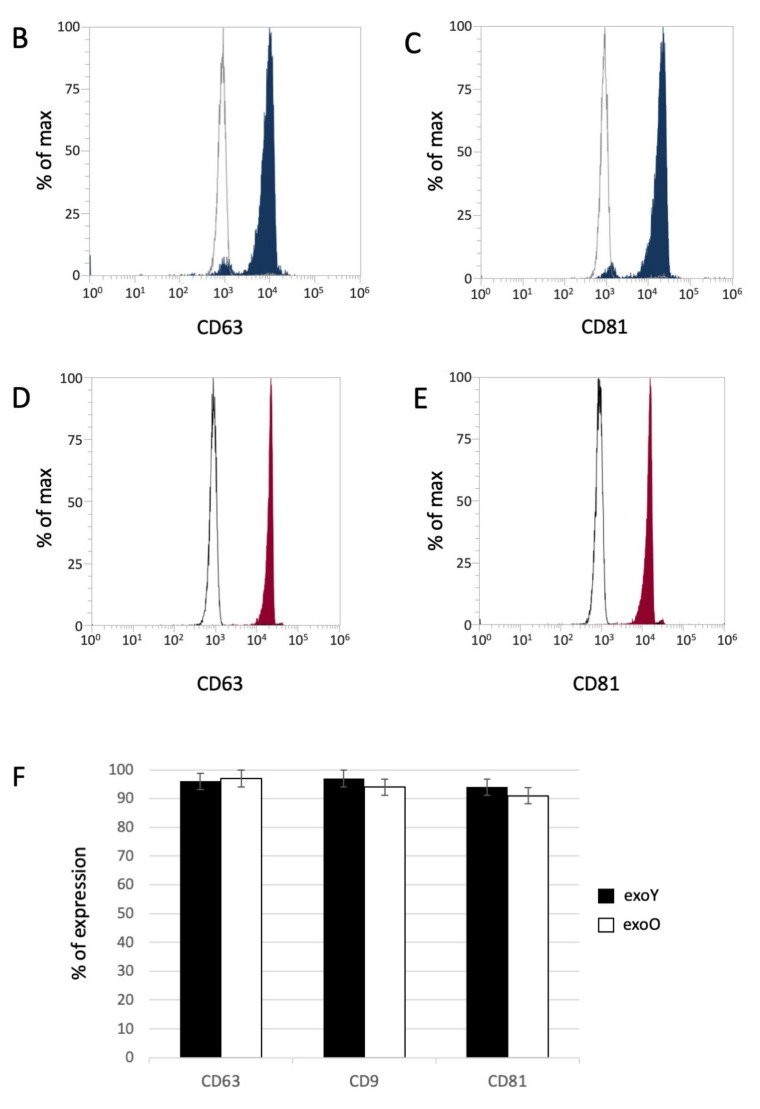

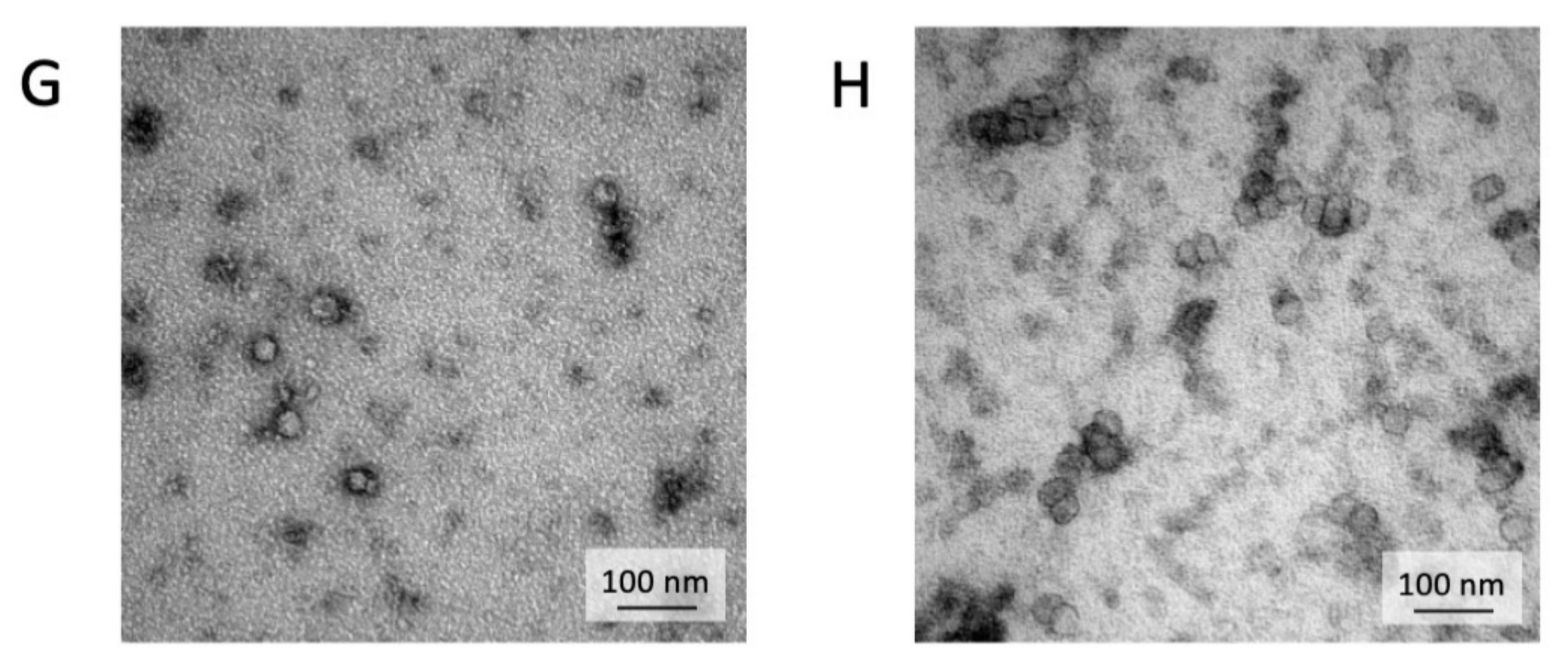
Exosome characterization: (**A**) average size of exosome diameters from young (exoY) and old (exoO) donors, (**B**) presence of CD63 on exoY, (**C**) presence of CD81 on exoY, (**D**) presence of CD63 on exoO, (**E**) presence of CD81 on exoO, **(F)** % of presence of specific markers of exosome derived from MSCs by gene expression; TEM of exosomes from young (**G**) and old (**H**) donors.

Exosomes were isolated by centrifugation and ultra- centrifugation from conditioned medium of dental pulp mesenchymal stem cells derived from young donors (exoY) and from older donors (exoO). Homogeneity and size of the purified exosomes were characterized by NanoSight using Nanoparticle tracking software (NTA) (Figure 1A, black bars for exoY, white bars for exoO) showed that the average size of the diameters of most EXOs ranged from 80 to 160 nm. NanoSight images and the presence of single peak size distribution profile indicate that the nano-sized vesicles isolated from the conditioned media of both classes of exosomes are highly purified (Figure 1). Cytofluorimeter analyses of specific exosome-related markers (i.e., CD63 and CD 81) revealed no difference between the two exosome types (exoY and O) as reported on Figure 1B–E. Molecular biology results identified exosomes-specific markers CD9, CD63, and CD81 in the analyzed exosomes (Figure 1F black bars for exoY, white bars for exoO). TEM demonstrated that EXOs presented as cup or round-shaped vesicles for exoY (Figure 1C) and for exoO (Figure 1D), respectively.

**Figure 2:**
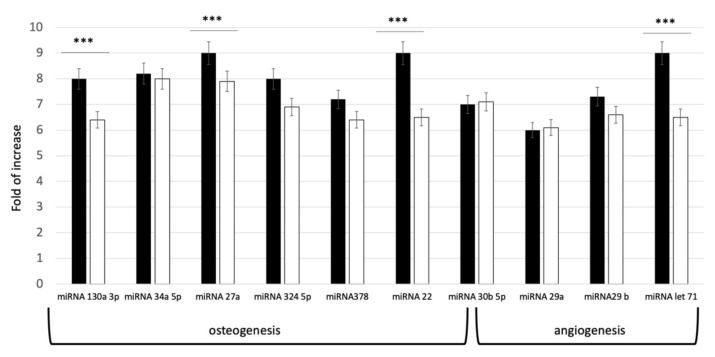
Expression levels of specific miRNA related to Osteogenesis and Angiogenesis pathways in young (black bar) and old (white bar) donors. *** *p* < 0.001.

In order to evaluate the commitment potential pathways defined by the isolated exosomes, a qPCR evaluation of miRNA content was performed. The miRNAs (miR-27a and miR-22, miR-130a-3p, miR-513b-5p, miR-30b-5p, miR-34a- 5p, miR-324-5p, and miR-378f) were selected, due to their effect on osteoblastic differentiation. For angiogenesis, let-7 and miR-29(a,b)-3p were investigated, since these miRNAs target genes regulating circulatory, blood vessel, cardiovascular system, vascular development and angiogenesis. As shown in Figure 2, all the selected miRNAs were expressed in both groups. Distinct levels of miRNA expression in the two groups were revealed. As reported on Figure 2, higher concentration of miRNAs was present indeed in the exosomes derived from younger donors (exoY).

### 3.2. EXO Influence on the Proliferation of MSCs

**Figure 3:**
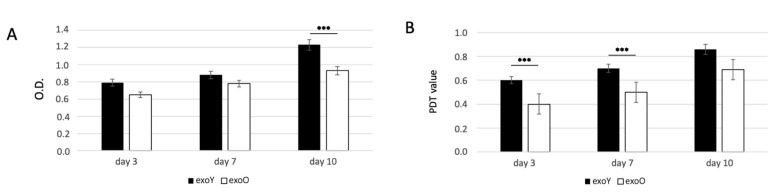
MTT (**A**) and PDT (**B**) assay of MSCs after treatment with exoY and exoO at 3, 7 and 10 days. *** *p* < 0.001.

Since the proliferation of MSCs is critical for tissue regeneration, we first investigated the effects of exoY and exoO on the proliferation of MSCs using MTT and PDT assays. The proliferative capacity of MSCs treated with exoY was significantly higher when compared with the group treated with exoO at day 3, day 5 and 10 (Figure 3A for MTT and Figure 3B for PDT).

### 3.3. EXO Influence Phenotypic Commitment of MSCs

**Figure 4:**
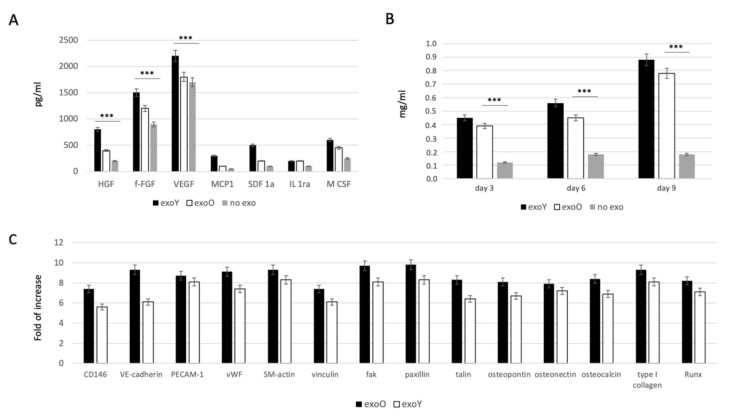
Evaluation of EXO influence on MSCs: (**A**) specifics growth factors quantification from MSCs after treatment with exoY and exoO compared to control (no exo). (**B**) ALP assay of MSCs under treatment with exoY, exoO compared to control (no exo). (**C**) qPCR of angiogenic and osteogenic factors from MSCs after treatment with exoY and exoO. *** *p* < 0.001.

In order to evaluate the effect of EXOs on MSC commitment, we investigated the ability of the cells to secrete specific growth factors related to immunomodulatory activity, such as monocyte chemotactic protein-1 (MCP-1), stromal cell-derived factor 1-alpha (SDF-1a), interleukin 1 receptor antagonist (IL-1ra) and macrophage colony-stimulating factor (M-CSF), and to angiogenic activity such as hepatocyte growth factor (HGF), basic fibroblast growth factor (b-FGF), vascular endothelial growth factor (VEGF). As reported in Figure 4A, the presence of both exosome types (from old and from young donors) induced an increase in paracrinal production from MSCs. Comparing the effect of the two groups, it is clear that the exosomes derived from younger donors induced a higher production of angiogenic factor, in particular VEGF and HGF, compared to the effect obtained from the old ones. Furthermore, osteogenic commitment was evaluated by means of alkaline phosphatase activity (ALP) as reported in Figure 4B. ALP activity was strongly more pronounced when MSCs were treated with exosomes but in this case, no clear difference was detected between two donor age groups. Angiogenic and osteogenic properties were in the end evaluated by means of qPCR as reported on Figure 4C, where it is clear the lower expression of factors related to endothelial phenotype, such as VEGF and bFGF, while the typical markers of osteoblasts, such as HGF and M-CSF, were significantly expressed.

## 4. Discussion

Stem cell-based therapies are currently successfully applied in regenerative medicine [16,33]. However, research is moving towards the development of cell-free alternatives, which allow the recruitment of endogenous stem cells, thus overcoming the limitations of cell transplantation. Additionally, it has to be noted that the biological capability of MSCs dramatically decreases in age-related disorders [33], as well in relation with the age of the donors [34]. Among cell-free approached, stem cell-derived exosomes are gaining popularity in regenerative medicine [35,36,37]. Indeed, previous investigations revealed therapeutic potential of stem cell-derived exosomes in numerous disorders such as myocardial ischemia/reperfusion injury, liver/renal failure, and traumatic brain/spinal cord injury [38].

Recent studies showed that epigenetic signals could be involved in the biological functions of MSCs and have an impact on exosomes, including oxygen concentration and aging.

The data here presented expands previous knowledge on donor age-related properties of MSCs. Several studies have shown that the microenvironment has a significant impact on stem cell activity [14,23,29,39]. Thus, we tested exosomes derived from donors of different age, considering as starting material SHEDs as “representant” of the young population and DPSCs as representant of aged donors. Our results exhibited that miRNAs related to osteogenesis and angiogenesis were expressed in both age groups. However, the levels of miRNA expression in exosomes derived from dental pulp stem cells of youngdonors was higher (Figure 2). Furthermore, cells treated with exoY exhibited a higher proliferation rate compared to the exoO group at all time points (Figure 3).

A decline in human DPSC function is observed with aging, and this could be a critical issue if applied to bone regeneration [14]. The aforementioned osteogenesis process is mediated by angiogenesis, in which blood supply induces osteoblast migration and bone mineralization. Therefore, also the angiogenetic commitment of stem cells is fundamental, as angiogenesis is closely associated with osteogenesis during the treatment of traumatic injuries and bone repair [12,32]. Our findings showed that the treatment of MSCs with exosomes derived from younger donor induced a higher production of angiogenic factor such as VEGF and HGF, compared with the exoO group (Figure 4A). In a study by Paino et al., stem cells from human dental pulp showed angiogenic potential as well as the ability to create vascularized woven bone tissue without the use of scaffolds [40]. Likewise, as revealed in Figure 4C, the expression of osteogenesis-related genes, such as ALP and RUNX2, was remarkably improved (FC > 2, *p* < 0.05) in the treatment group with exoY (Figure 4C).

The present study demonstrates that dental pulp derived-exosomes from both young and old donors promoted the proliferation and differentiation of MSCs, however a more pronounced effect was observed in the younger group. Hence, exosomes derived from the dental pulp have the potential to be utilized for the treatment of cranio-maxillofacial bone defects. Taking into account the limitations of this study, a conspicuous trend is observed between the expression levels of specific osteogenic and angiogenic-related miRNAs and the expression of osteogenic- and angiogenic-related genes of exosome-treated MSCs. The osteogenic and pro-angiogenic effects of SHED-derived exosomes as well as the underlying mechanisms possibly mediated by the shuttled miRNAs warrant further investigations.

## 5. Conclusions

As we reported previously for DPSCs, we have found that exosomes derived from the dental pulp have an impressive osteogenic potential. The microenvironment of the bone marrow contains growth factors and exosomes that may contribute to bone regeneration by enhancing angiogenesis. Furthermore, as we age, bone density declines because of oxidative stress and reduced MSC availability within the bone marrow microenvironment.

As a result of our research, exosomes can also be considered as novel cell-free therapy, alone or in combination with scaffolds, used as bioactive agents for bone regeneration.

## Figures and Tables

**Table 1:** Primers.

Gene	FOR	REV	Product (bp)
vWF	*ATGCTCCTCACGTATGGTC*	*TGTGTGGGATC GACAAGACAC*	179
SM-actin	*TGGGAGAGCATTT TCCAGCCAACTCA*	*CATACGATCACCATCC*	133
vinculin	*GCTAACTTAGTGCTTTGCATGTGTCTT*	*AGTCTTAGTCACC ACAGGAACCA*	132
fak	*GCAGCGAGGAGCAGAGCGACAC*	*TAGTGAAGAGAC CCTA*	112
paxillin	*CATCATTGGCTGGAAAGCGAG*	*GAGTTGAATGG GCTCATTGCTCT*	192
talin	*ACCAGTCTCCAGATCGAGATGTT*	*GCAGA TGAGCCAGCA*	155
osteopontin	*GTGGTAGGTGATGTGGAGAAGAAA*	*CTCTGGGA CAGCAAGAGCAA*	183
PECAM-1	*CTTGTCACAGGAGATCACA*	*GAGTATGCCAA TCCT*	149
VE-cadherin	*CTGGGTAGATATGGCCTGGGTGA*	*TTCAAGAGA GATGAGAGTGTGAG*	148
Runx	*CCGAGACCAATCATTGCGGTCG*	*AGGTGAAGACTG CCTGTG*	163
type I collagen	*CCGAGACCAAGGTGAAGACTG CCTGA*	*CATTGCGGTCGTG*	156
osteocalcin	*CCGAGACCAAGGTGAAGGCGGTCGT*	*GACTG CCTGATCATT*	193
osteonectin	*CCGAGACCAAGATTG*	*CGGTCGTGGTGAAGACTG CCTGT*	163
